# Effect of different bolus application methods on target dose in radiotherapy after radical mastectomy

**DOI:** 10.3389/fonc.2026.1806104

**Published:** 2026-04-13

**Authors:** Xinyu Zhao, Wen Wang, Feng Wang, Hao Su, Xiaojuan Sun

**Affiliations:** Department of Radiation Oncology, Binzhou Medical University Hospital, Binzhou, Shandong, China

**Keywords:** bolus, breast neoplasms, intensity-modulated radiation therapy (IMRT), volumetric-modulated arc therapy (VMAT), dosimetry, radiotherapy

## Abstract

**Objective:**

To assess the impact of bolus addition methods on the target dose of radiotherapy following radical mastectomy for breast cancer.

**Methods:**

Thirty patients who underwent radiotherapy after radical mastectomy between January and August 2023 were included in this study. Each patient underwent two CT scans: CT1 (no bolus) and CT2 (with bolus placed on the chest wall). Using the CT1 images, a virtual bolus was added to create a radiotherapy Plan1 that met clinical and dosimetric requirements. Plan2 was generated from the CT2 images with the added bolus, and Plan3 was derived by copying Plan1 to CT2 to simulate the dose distribution during treatment. Radiotherapy plans were developed using volumetric-modulated arc therapy (VMAT) and intensity-modulated radiotherapy (IMRT) techniques. The dosimetric parameters for the target volume and organs at risk (OAR) were analyzed for each plan.

**Results:**

Statistically significant differences were observed in the homogeneity index (HI) and conformity index (CI) of the target volume when comparing Plan3 to Plan1 and Plan2 in both VMAT and IMRT plans (P < 0.05). Additionally, significant differences in V_107%_, CI, and monitor units were found when comparing VMAT to IMRT across all three plans (P < 0.05). No significant differences in OAR doses were found with the VMAT plan. However, the IMRT plan showed significant differences in heart V_30_ between Plan1 and Plan2 and Plan3 (P < 0.05). Significant differences were also observed between VMAT and IMRT for lung V_5_, lung V_30_, esophagus D_mean_, humeral head D_mean_, and uninjured breast D_mean_ across all three plans (P < 0.05).

**Conclusion:**

The addition of a bolus during radiotherapy planning provides a more accurate reflection of the dose distribution in the target area and the dose received by OARs. The VMAT plan achieved better target conformity and significantly reduced monitor units, potentially shortening treatment time and improving machine efficiency.

## 1 Introduction

Malignant breast tumors are the leading cause of cancer-related deaths among women (1, 2). In recent years, advancements in surgical techniques and systemic therapies have significantly improved breast cancer treatment outcomes (3–5). Radiotherapy has played a crucial role in these improvements, enhancing local control and overall survival rates (6, 7). However, inadequate and uneven dose distribution to the chest wall following modified radical mastectomy can increase the risk of recurrence (8). Given the critical role of the superficial lymphatic network in tumor spread, preventing skin recurrence should not be compromised solely for target dose accuracy (9).

The dose buildup effect results in uneven irradiation dose to the superficial target area, potentially compromising therapeutic effectiveness (10, 11). A bolus is commonly applied to the chest wall to compensate for dose buildup, ensuring adequate dose delivery to both the chest wall and the skin (8). Bolus application typically increases skin dose by 20–30%, significantly reducing the risk of chest wall recurrence (12, 13).

Despite its importance, no standardized guidelines exist for selecting bolus materials, determining thickness and coverage, or deciding the frequency of application. Historically, materials such as paraffin, petrolatum gauze, polystyrene, and commercial compensators were used (14, 15). However, these traditional methods often resulted in poor skin adhesion and air gaps, leading to dose deviations during radiotherapy (16, 17). Recently, 3D-printed boluses have garnered attention (18), but their complexity and time-consuming production process make them less practical than boluses made from self-adhesive biopolymers. These advanced compensators, composed of solid paraffin, low-density polyethylene, and styrene-maleic anhydride copolymer, offer excellent adhesion and are cost-effectiveness. Bolus thickness significantly affects skin dose. Studies indicate that increasing bolus thickness exacerbates skin reactions, with pronounced toxic effects observed at thicknesses exceeding 5 mm (19–22). Daily bolus application can intensify these side effects, disrupting treatment and potentially causing patients to discontinue therapy, thus compromising efficacy. Optimizing application frequency is therefore essential (23, 24).

From a dosimetric perspective, retracting PTV 3 mm subcutaneously during treatment planning helps ensure proper dose distribution (25). Applying a 5-mm bolus improves target dose uniformity and minimizes the risk of missed areas (13).

Currently, there are no clear guidelines for bolus application in clinical practice. Two primary methods are generally used: 1. Adding the bolus during the CT simulation: This approach allows the bolus to be adjusted, trimmed, or shaped to improve skin fit and minimize air gaps. These adjustments reduce uncertainties in the treatment plan and enhance dose accuracy; 2. Adding the bolus during treatment: In this method, the bolus is applied directly during radiotherapy sessions based on clinical requirements. A virtual bolus is planned during the CT simulation, and an accurate bolus is applied during treatment to increase the chest wall skin dose. This approach ensures precise coverage of high-risk areas for skin recurrence while maintaining flexibility. When choosing between these methods, it is essential to weigh the differences in dose distribution resulting from using a virtual bolus during planning versus applying a physical bolus during treatment.

For this purpose, a 5-mm thick virtual bolus is typically used in radiotherapy planning. However, discrepancies can occur between the planned and actual dose distributions when the bolus covers a 30 cm × 30 cm area on the patient’s surface. This study aims to investigate the dosimetric impact of using a bolus in breast cancer radiotherapy and to identify strategies for optimizing treatment planning.

## 2 Materials and methods

### 2.1 General information

Thirty patients who underwent modified radical mastectomy for breast cancer between January and August 2023 were randomly selected, and their CT images were collected. Inclusion Criteria: A confirmed diagnosis of breast cancer based on postoperative pathology; underwent modified radical mastectomy; normal cardiopulmonary function; signed informed consent for radiotherapy; and approval from the hospital’s ethics committee. Exclusion Criteria: Inability to tolerate lying flat for extended periods; impaired upper extremity function, including limited lifting and abduction; and poor wound healing at the surgical site.

### 2.2 Positioning and CT image acquisition

All patients were positioned supine on a carbon fiber body frame with their arms crossed and holding frame handles for fixation, using a thermoplastic mask. Prior to imaging, marker lines were drawn on the patient’s skin to fix the relative position of the patient and the frame. Once the body mask was secured, three cross marks were placed on a skin area near the target region, chosen for its low mobility, and three metal reference points were marked.

CT imaging was performed using a Philips large-bore CT scanner. Each patient underwent two CT scans: CT1 (without a bolus) and CT2 (with a bolus applied). The scans typically covered from the annulus to the lower edge of the liver, with a slice thickness of 5 mm. The acquired CT images were transferred to the Eclipse treatment planning system (TPS).

### 2.3 OAR contouring and radiotherapy plan design

The target area was delineated based on the International Commission on Radiation Protection Report 83 guidelines (26). OARs included the heart, lungs, uninjured breast tissue, esophagus, affected humeral head, and spinal cord. A single physician performed the target delineation, with review and confirmation by a senior physician.

Treatment planning was carried out using the Eclipse TPS, and all patients were treated with the Varian Trilogy linear accelerator. VMAT and IMRT were used to optimize treatment delivery. The final treatment plan was selected based on a combination of dosimetric parameters, including dose uniformity, conformity, and minimizing OAR dose exposure. The prescribed dose was 50 Gy in 25 fractions (5 per week), ensuring that 95% of the Planning Target Volume (PTV) received the prescribed dose. Dose constraints for OARs included: Affected lung: V_5_ < 60%, V_20_ < 30%, V_30_ < 20%; Whole lung: D_mean_ < 15 Gy; Heart: V30 < 5%; Uninjured breast: D_mean_ < 5 Gy.

The isocenter for all radiation fields was placed at the geometric center of the PTV, and the field arrangements were designed to minimize irradiation of lung tissue and other critical structures. Each patient underwent six treatment plans: Plan1-VMAT, Plan1-IMRT, Plan2-VMAT, Plan2-IMRT, Plan3-VMAT, Plan3-IMRT. Plan Details: Plan1: A virtual bolus was added to the CT1 scan, creating a treatment plan without a physical bolus. A 5-mm thick virtual bolus was manually added to the chest wall, with a density of 1.0 g/cm³. Plan2: A physical bolus was added during the CT scan to generate treatment plans from CT2. The virtual bolus from Plan1 was omitted. Plan3: This plan used a virtual bolus during planning and an accurate physical bolus during treatment. Image fusion technology was employed to transfer target areas and OARs from CT1 to CT2, removing the virtual bolus in CT2. Plan verification was done by directly transferring dosimetric parameters from Plan1 to CT2.

### 2.4 Evaluation of radiotherapy plans

According to ICRU Report 83, dosimetric parameters were evaluated using dose-volume histograms (DVH). Parameters for the target volume included: D_2%_, D_50%_, D_98%_ (doses received by 2%, 50%, and 98% of the target volume, respectively); V_95%_, V_107%_ (volumes receiving 95% and 107% of the prescribed dose, respectively); HI and CI. For OARs, the following parameters were evaluated: affected lung V_5_, V_20_, V_30_, D_mean_; heart V_30_, D_mean_; spinal cord D_max_; esophagus D_mean_; affected humeral head D_mean_; uninjured breast D_mean_. The total number of monitor units (MUs) for each plan was also recorded.

### 2.5 Statistical methods

Data were analyzed using SPSS 23.0 software. The normality of the data was first assessed. For data that did not follow a normal distribution, results were presented as the median (M) and interquartile range (P25, P75). The Wilcoxon signed-rank test was used for pairwise comparisons, while the Kruskal-Wallis H test was employed to analyze multiple groups. A p-value of less than 0.05 was considered statistically significant.

## 3 Results

### 3.1 Comparison of dosimetric parameters in the target area

**Figure 1:**
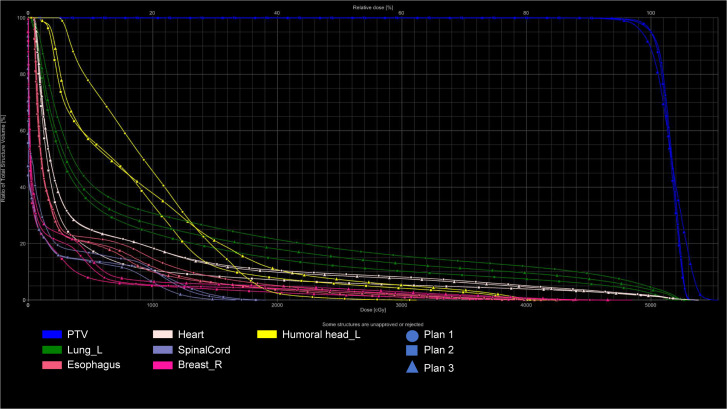
DVH of target volumes and OARS: Three Radiotherapy Plans (IMRT).

**Figure 2:**
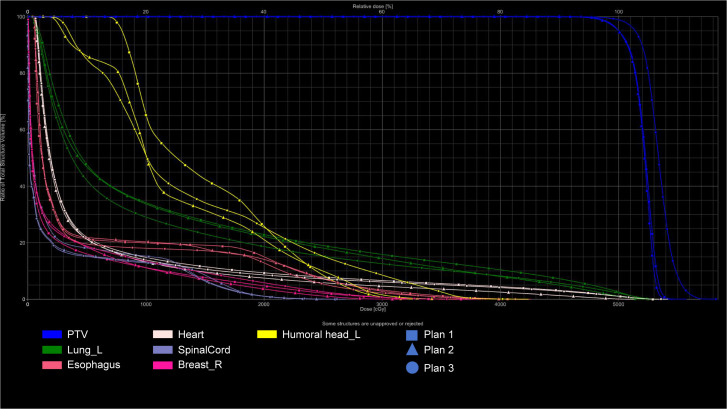
DVH of target volumes and OARS: Three Radiotherapy Plans (VMAT).

All VMAT and IMRT plans (Plan1, Plan2, Plan3) met the clinical dosimetric requirements. Detailed data are presented in **Figures 1** and **2**.

**Figure 3:**
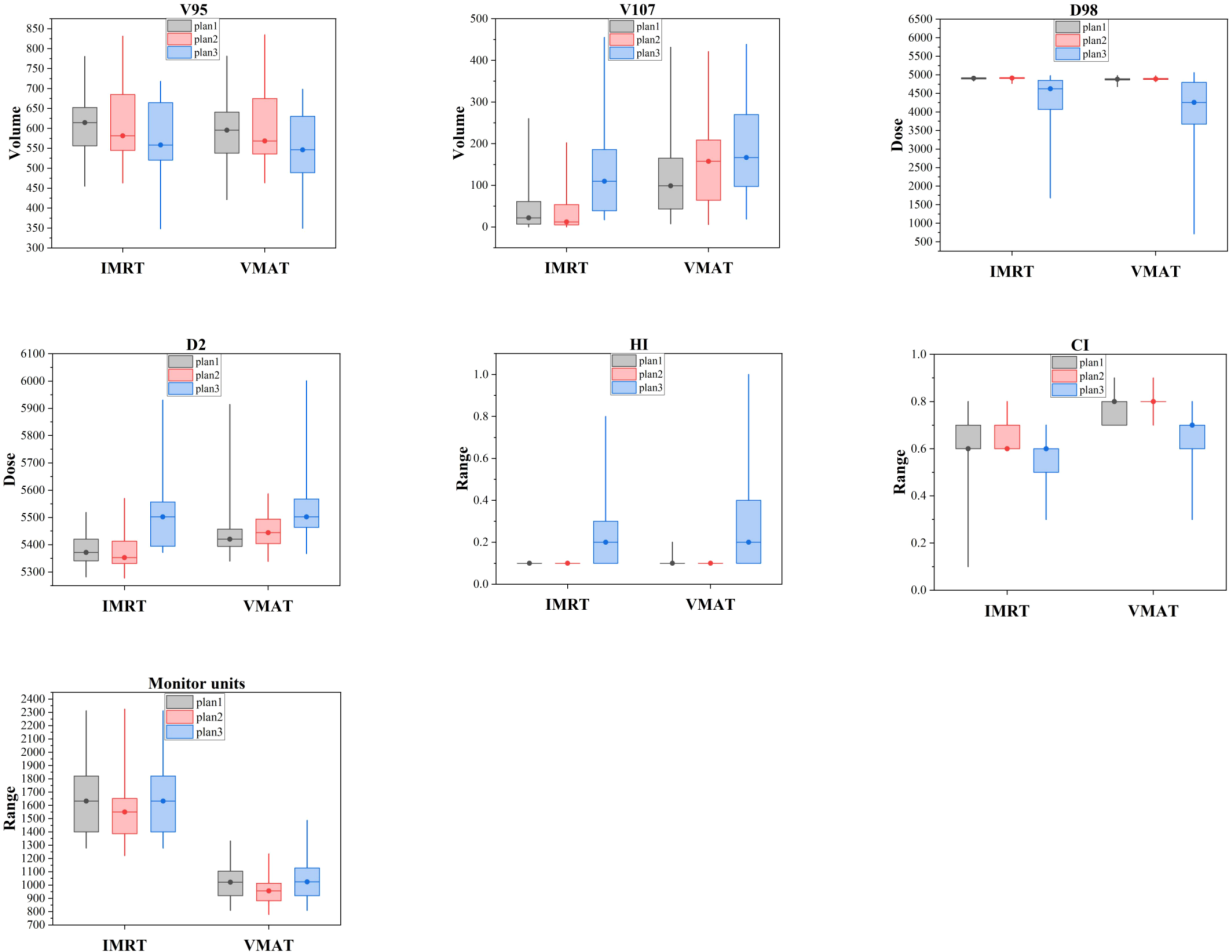
Dose parameters of the target area for VMAT and IMRT across three different planning methods.

In the VMAT plans, Plan1 and Plan2 showed significantly better HI and CI than Plan3 (P < 0.05). Furthermore, Plan2 required fewer monitor units than Plan1 and Plan3 (P < 0.05). The V_107%_ value for Plan3 was higher than that of Plan1 (P < 0.05). Detailed data are presented in **Figure 3**; **Table 1**.

**Table 1:** Comparison of dosimetric parameters under three different VMAT plans *M (P25, P75*).

Dose parameters	Plan1-VMAT	Plan2-VMAT	Plan3-VMAT	P	P12	P13	P23
V_95%_(cm^3^)	595.5(537.0,644.3)	568.5(535.0,676.1)	546.3(488.7,632.6)	0.167	0.767	0.089	0.124
V_107%_(cm^3^)	98.7(41.1,166.5)	157.5(53.7,210.8)	166.7(93.5,275.0)	0.063	0.280	0.017	0.243
D_98%_(Gy)	48.8(48.6,49.0)	48.9(48.7,49.0)	42.6(36.3,48.0)	0.000	0.371	0.000	0.000
D_2%_(Gy)	54.2(53.9,54.6)	54.4(54.0,54.9)	55.0(54.6,55.7)	0.000	0.225	0.000	0.001
HI	0.1(0.1,0.1)	0.1(0.1,0.1)	0.2(0.1,0.4)	0.000	0.317	0.000	0.000
CI	0.8(0.7,0.8)	0.8(0.8,0.8)	0.7(0.6,0.7)	0.000	0.036	0.000	0.000
Monitor units(MU)	1022.0(918.0,1111.0)	956.5(880.0,1015.0)	1024.5(918.0,1132.0)	0.018	0.014	0.871	0.015

P indicates the P value of the comparison between the three groups of Plan1, Plan2, and Plan3; P12 indicates the P value of the comparison between Plan1 and Plan2; p13 indicates the P value of the comparison between Plan1 and Plan3; P23 indicates the P value of the comparison between Plan2 and Plan3.

For the IMRT plans, Plan3 exhibited a higher V_107%_ value compared to Plan1 and Plan2, while the HI and CI values for Plan1 and Plan2 were superior (P < 0.05), as shown in **Figure 3**; **Table 2**.

**Table 2:** Comparison of dosimetric parameters under three different IMRT plans *M* (*P25, P75*).

Dose parameters	Plan1-IMRT	Plan2-IMRT	Plan3-IMRT	P	P12	P13	P23
V_95%_(cm^3^)	614.5(555.9,653.5)	581.5(543.1,685.3)	558.4(519.5,665.8)	0.377	0.779	0.201	0.274
V_107%_(cm^3^)	21.8(6.0,63.5)	12.0(4.5,60.5)	109.6(38.6,187.5)	0.000	0.610	0.001	0.000
D_98%_(Gy)	49.1(48.9,49.2)	49.1(49.0,49.3)	46.3(40.6,48.6)	0.000	0.234	0.000	0.000
D_2%_(Gy)	53.7(53.4,54.3)	53.5(53.3,54.1)	55.0(53.9,55.6)	0.000	0.469	0.000	0.000
HI	0.1(0.1,0.1)	0.1(0.1,0.1)	0.2(0.1,0.3)	0.000	1.000	0.000	0.000
CI	0.6(0.6,0.7)	0.6(0.6,0.7)	0.6(0.5,0.6)	0.000	0.444	0.003	0.000
Monitor units(MU)	1632.5(1396.0,1827.0)	1550.0(1381.0,1669.3)	1632.5(1396.0,1827.0)	0.429	0.261	1.000	0.261

When comparing VMAT and IMRT across all plans (Plan1, Plan2, and Plan3), the V_107%_ was higher for VMAT, and VMAT also showed superior CI and monitor units (P < 0.05). Detailed results are shown in **Figure 3**; **Table 3**.

**Table 3:** Comparison of dosimetric parameters between VMAT and IMRT for three different planning methods *M(P25, P75)*.

Dose parameters	Plan1		Plan2		Plan3		P1	P2	P3
	Plan1-VMAT	Plan1-IMRT	Plan2-VMAT	Plan2-IMRT	Plan3-VMAT	Plan3-IMRT			
V_95%_(cm^3^)	595.5(537.0,644.3)	614.5(555.9,653.5)	568.5(535.0,676.1)	581.5(543.1,685.3)	546.3(488.7,632.6)	558.4(519.5,665.8)	0.695	0.620	0.287
V_107%_(cm^3^)	98.7(41.1,166.5)	21.8(6.0,63.5)	157.5(53.7,210.8)	12.0(4.5,60.5)	166.7(93.5,275.0)	109.6(38.6,187.5)	0.000	0.000	0.029
D_98%_(Gy)	48.8(48.6,49.0)	49.1(48.9,49.2)	48.9(48.7,49.0)	49.1(49.0,49.3)	42.6(36.3,48.0)	46.3(40.6,48.6)	0.000	0.000	0.124
D_2%_(Gy)	54.2(53.9,54.6)	53.7(53.4,54.3)	54.4(54.0,54.9)	53.5(53.3,54.1)	55.0(54.6,55.7)	55.0(53.9,55.6)	0.008	0.000	0.308
HI	0.1(0.1,0.1)	0.1(0.1,0.1)	0.1(0.1,0.1)	0.1(0.1,0.1)	0.2(0.1,0.4)	0.2(0.1,0.3)	0.317	1.000	0.101
CI	0.8(0.7,0.8)	0.6(0.6,0.7)	0.8(0.8,0.8)	0.6(0.6,0.7)	0.7(0.6,0.7)	0.6(0.5,0.6)	0.000	0.000	0.005
Monitor units(MU)	1022.0(918.0,1111.0)	1632.5(1396.0,1827.0)	956.5(880.0,1015.0)	1550.0(1381.0,1669.3)	1024.5(918.0,1132.0)	1632.5(1396.0,1827.0)	0.000	0.000	0.000

### 3.2 Comparison of dosimetric parameters for OARs

In the VMAT plans, no significant differences in doses to the OARs were observed. However, in the IMRT plans, Plan1 showed a statistically significant difference in heart V_30_ compared to Plan2 and Plan3 (P < 0.05), as shown in **Table 4**.

**Table 4:** Comparison of OARs dose for VMAT and IMRT under three different planning methods *M(P25, P75)*.

OARs parameters	Plan1		Plan2		Plan3		P1	P2	P3
	Plan1-VMAT	Plan1-IMRT	Plan2-VMAT	Plan2-IMRT	Plan3-VMAT	Plan3-IMRT			
Lung V_5_(%)	50.1(47.4,53.2)	42.1(40.7, 47.0)	49.5(47.6,52.0)	43.0(40.7, 47.7)	52.2(45.4,53.7)	44.4(40.7, 47.5)	0.000	0.000	0.000
Lung V_20_(%)	22.9(20.5,24.4)	21.7(20.9, 26.2)	22.2(20.6,23.9)	22.2(19.9, 26.0)	22.2(18.4,27.2)	23.7(20.3, 27.6)	0.888	0.717	0.442
Lung V_30_(%)	15.3(13.4,16.8)	17.3(16.1, 21.1)	14.9(12.4,16.8)	17.7(15.2, 20.7)	15.8(11.9,20.4)	19.0(15.4, 22.4)	0.000	0.001	0.029
Lung D_mean_(Gy)	12.4(11.6,13.0)	12.3(11.6, 13.8)	12.1(11.2,12.8)	12.3(11.3, 13.8)	12.5(10.4,14.7)	13.1(11.3, 14.9)	0.574	0.318	0.329
Heart V_30_(%)	2.5(0.0,4.8)	3.5(0.0, 6.6)	1.4(0.0,4.3)	2.9(0.0, 5.5)	1.5(0.0,4.4)	2.5(0.0, 7.1)	0.297	0.454	0.577
Heart D_mean_(Gy)	4.3(1.4,5.8)	4.3(1.0, 6.1)	3.9(1.3,5.4)	3.4(1.0, 5.2)	3.6(1.4,5.5)	3.6(1.1, 5.8)	0.496	0.496	0.690
Spinal cord D_max_(Gy)	30.3(25.0,33.5)	24.2(19.3, 28.6)	31.6(26.1,33.3)	22.9(19.5, 27.5)	29.5(26.2,32.9)	26.2(16.2, 32.2)	0.003	0.000	0.069
Esophagus D_mean_(Gy)	6.9(5.3,8.7)	4.3(3.5, 6.2)	6.7(5.1,7.9)	4.6(3.1, 6.0)	6.7(4.4,8.7)	4.0(3.3, 6.0)	0.000	0.000	0.002
Humerus D_mean_(Gy)	17.3(13.2,26.3)	6.6(4.4,8.9)	17.3(10.4,26.1)	6.1(3.9,8.3)	15.2(11.7,22.2)	6.3(3.7,9.5)	0.000	0.000	0.000
Uninjured breast D_mean_(Gy)	3.2(2.5,4.0)	1.20(0.9, 1.7)	3.7(2.7,4.3)	1.2(0.8, 1.8)	3.9(2.8,4.9)	1.4(1.0, 2.4)	0.000	0.000	0.000

**Figure 4:**
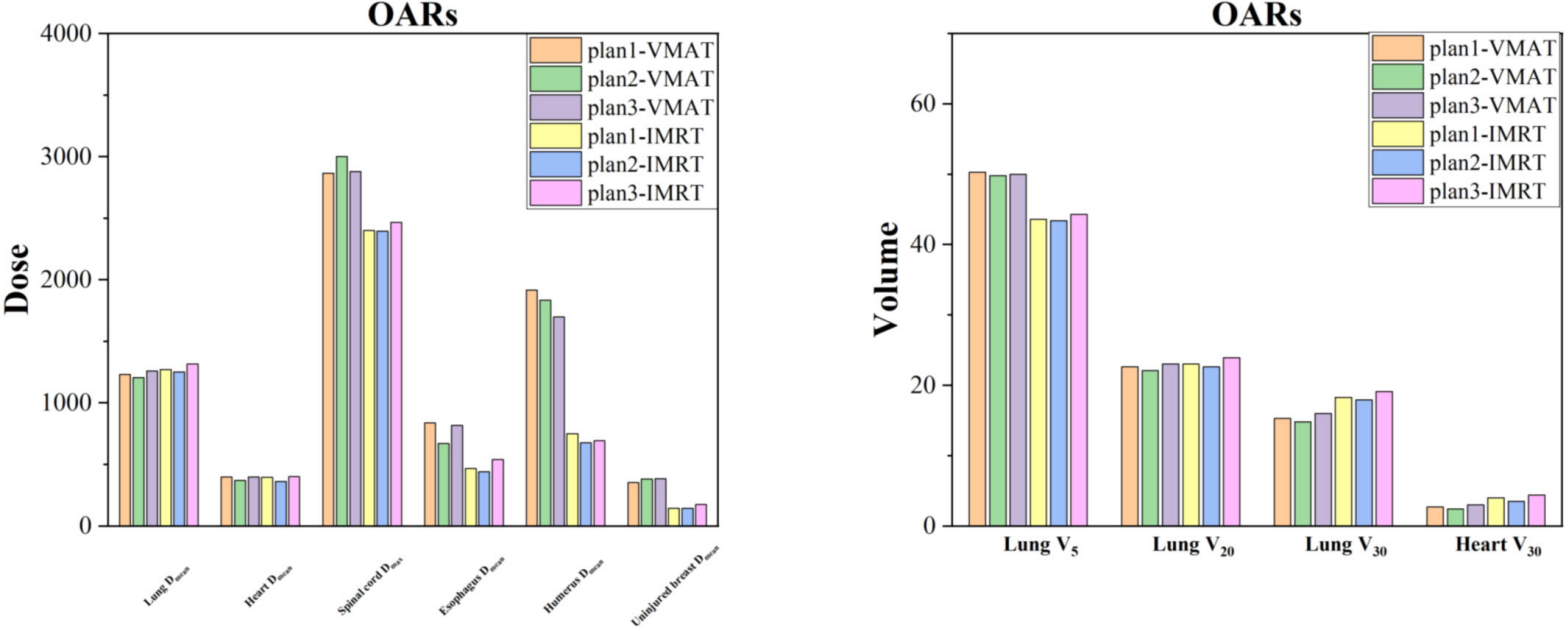
Dose comparison of OARs for VMAT and IMRT across three different planning methods.

For VMAT, no significant differences in lung D_mean_, lung V_20_, heart D_mean_, or heart V_30_ were observed compared to IMRT. In contrast, for Plan1, Plan2, and Plan3, the lung V5, esophagus D_mean_, humeral head D_mean_, and uninjured breast D_mean_ doses were higher for VMAT than for IMRT (P < 0.05). Additionally, for Plan1 and Plan2, the spinal cord D_max_ was greater for VMAT than IMRT (P < 0.05). Detailed information is presented in **Table 4**; **Figure 4**.

## 4 Discussion

Most research on bolus application in breast cancer has focused on its impact on prognosis, with less emphasis on its dosimetric impact in modern techniques such as IMRT and VMAT (27–29). This study primarily explored bolus application in these contexts and reached two main conclusions: (1) the optimal approach is to apply the bolus during CT simulation; (2) if bolus application before CT positioning is not feasible, VMAT should be prioritized to minimize errors from improper bolus use. Plan2, with a physical bolus applied after CT, demonstrated superior conformity and treatment efficiency in VMAT and IMRT. The use of virtual bolus in Plan1 led to discrepancies in dose calculations due to differences in the density and shape of the bolus. In particular, the appearance of air gaps can lead to differences between the two when the TPS calculates the dose (30). When comparing VMAT and IMRT, VMAT’s advantages were most apparent in target conformity and delivery efficiency, suggesting that VMAT minimizes errors related to bolus application.

In recent years, VMAT and IMRT have become the mainstream radiotherapy techniques for treating breast cancer (31–33). Chen et al. (32) compared various treatment modalities, including 3D conformal, 3D conformal in-field, conformal intensity modulation, volumetric intensity modulation, electron beam compensation, and hybrid approaches. Their dosimetric evaluation indicated that conformal intensity modulation offered the greatest advantages in target conformity and OARs protection. VMAT typically delivers more homogeneous doses, improves conformity, reduces the mean lung dose, decreases monitor units (MUs), and shortens treatment time. In contrast, IMRT is more effective in protecting healthy lung and breast tissue, but it does not significantly reduce the radiation dose to the affected lung (34–36).

This study found that the V_107%_ value was smaller in Plan1 (without bolus) compared to Plan3 (with bolus). This discrepancy was likely due to variations in the two CT scans, such as setup errors and respiratory motion, which affect thoracic and lung volumes and, subsequently, the delineation of the target area. However, Plan3, which used a verification method, replicated Plan1 onto CT2 without the need for dose optimization, directly yielding corresponding dosimetric parameters. To meet the 95% prescription dose requirement for the target area, the overall target dose was increased, which led to a higher V_107%_ in Plan3, as shown in the DVH.

In comparing the dose to OARs, we found that in the IMRT plan, the heart V_30_ dose in Plan1 was greater than in Plan2, suggesting that using CT images with bolus positioning for IMRT could reduce the heart V_30_ dose. However, this trend was not observed in the VMAT plan, indicating that the presence or absence of bolus for positioning had minimal impact on heart dose in the VMAT plans. For lung doses, the VMAT plan showed a greater lung V_5_ dose on the affected side compared to IMRT, but a lower lung V_30_ dose. Additionally, the VMAT plan showed higher doses to the spinal cord D_max_ and other OARs compared to IMRT, though all doses to OARs remained within safe limits. Therefore, the main advantage of the VMAT plan in this study was its ability to protect the heart and reduce the lung V_30_ doses.

This study had several limitations. First, it was a retrospective analysis. Second, there were inherent differences between the two CT scans, particularly in Plan3, which involved using image fusion technology to transfer the contouring from CT1 to CT2. While the discrepancies between the two scans contributed to the overall positioning errors, when combined with treatment-level considerations, these limitations were deemed acceptable for the study’s scope. Third, the influence of air gaps on the plan was ignored in the course of this study. The self-adhesive bolus used in the study has good adhesion and produces very little or almost no air gaps. A key strength of this study was its clear guidance for physicists on bolus application, highlighting that adding a bolus before CT positioning is the most reasonable approach. However, this requires careful planning in advance. For patients with specific positioning needs, the necessity for repositioning after adding a bolus could be overlooked, leading to potential complications. In large tumor centers, conducting two separate CT localizations can reduce operational efficiency and increase patient costs. Consequently, VMAT plans demonstrated a significant advantage in target conformity and machine utilization when planning CT images without a bolus.

## Data Availability

The original contributions presented in the study are included in the article/supplementary material, further inquiries can be directed to the corresponding author/s.
